# *Hoxd10* Is Required Systemically for Secretory Activation in Lactation and Interacts Genetically with *Hoxd9*

**DOI:** 10.1007/s10911-020-09454-3

**Published:** 2020-07-23

**Authors:** John D. Landua, Ricardo Moraes, Ellen M. Carpenter, Michael T. Lewis

**Affiliations:** 1 Department of Molecular and Cellular Biology, Lester and Sue Smith Breast Center, Dan L Duncan Cancer Center, Baylor College of Medicine, One Baylor Plaza, Room N1210; BCM600, Houston, TX 77030 USA; 2 Center for Cell and Gene Therapy, Texas Children’s Feigin Center, Baylor College of Medicine, 1102 Bates Avenue, Houston, TX 77030 USA; 3 Division of Undergraduate Education, National Science Foundation, 2415 Eisenhower Avenue, Alexandria, VA 22314 USA

**Keywords:** Homeobox gene, Lactogenesis, Milk secretion, Lactation failure, Alveolar development

## Abstract

Targeted disruption of the murine *Hoxd10* gene *(ΔHoxd10)* leads to a high frequency of localized (gland-to-gland or regionally within a gland) lactation impairment in homozygous mutant mice as a single gene mutation. The effect of *Hoxd10* disruption was enhanced by simultaneous disruption of *Hoxd9 (ΔHoxd9/d10)*, a mutation shown previously to have no effect on mammary function as a single gene alteration. Mammary glands of homozygous *ΔHoxd10* and *ΔHoxd9/d10* females were indistinguishable from those of wild type littermate and age-matched control mice in late pregnancy. However, in lactation, 47% of homozygous *ΔHoxd10* females, and 100% of homozygous *ΔHoxd9/d10* females, showed localized or complete failure of two or more glands to undergo lactation-associated morphological changes and to secrete milk. Affected regions of *ΔHoxd10* and *ΔHoxd9/d10* mutants showed reduced prolactin receptor expression, reduced signal transducer and activator transcription protein 5 (STAT5) phosphorylation, reduced expression of downstream milk proteins, mislocalized glucose transporter 1 (GLUT1), increased STAT3 expression and phosphorylation, recruitment of leukocytes, altered cell cycle status, and increased apoptosis relative to unaffected regions and wild type control glands. Despite these local effects on alveolar function, transplantation results and hormone analysis indicate that *Hoxd10* primarily has systemic functions that confer attenuated STAT5 phosphorylation on both wild type and *ΔHoxd10* transplants when placed in *ΔHoxd10* hosts, thereby exacerbating an underlying propensity for lactation failure in C57Bl/6 mice.

**Electronic supplementary material:**

The online version of this article (10.1007/s10911-020-09454-3) contains supplementary material, which is available to authorized users.

## Introduction

The mouse mammary gland is a powerful model system for the study of cellular differentiation and gene function in organ development at the molecular, cellular, organ and organismal levels [1, 2]. Development of the mammary gland is primarily post-pubertal, and can be characterized as a series of morphological and functional transitions, or switches, in which critical developmental decisions are made concerning cell identity, cell fate, pattern formation, and differentiation [3–5]. These transitions are under both local and systemic control.

Ductal development in the mammary gland begins during embryogenesis with the formation of a rudimentary ductal tree [6–8]. At puberty, systemic ovarian hormones stimulate rapid and invasive ductal elongation and branching morphogenesis. Upon reaching the limits of the fat pad, ductal elongation ceases and, unless stimulated by pregnancy, the ductal tree becomes relatively growth quiescent. While there is a small amount of milk protein synthesis in the virgin animal, the gland is neither morphologically nor functionally differentiated to secrete milk.

Systemic hormonal changes during pregnancy (e.g. estrogen, progesterone, prolactin, and glucocorticoids) initiate a transition from a predominantly ductal to a predominantly lobuloalveolar gland morphology [9, 10]. Near mid-pregnancy, the alveolar epithelium increases its capacity to synthesize milk proteins and acquires the ability to accumulate cytoplasmic lipid droplets. However, secretion of protein and lipid is inhibited by high systemic levels of progesterone. At parturition, progesterone levels fall rapidly in the face of elevated prolactin levels inducing the gland to undergo secretory activation and secrete large quantities of milk. It is only with the passage through secretory activation that the mammary gland can be considered functionally differentiated.

Homeobox genes act as critical regulators of cell identity and cell fate during development of many organisms [11–15]. In mammals, over 100 homeobox genes have been identified that comprise multiple gene families [16]. Individual members of many of these gene families are known to be expressed in the mammary gland, or in cultured mammary epithelial cell lines [17, 18]. Notable among the homeobox gene families is the *Hox* family, which is comprised of all genes in the *Hox* complex. In the mouse (and human), 39 *Hox* complex genes are arranged in four paralogous gene clusters, *Hoxa*, *Hoxb*, *Hoxc*, and *Hoxd*, one on each of four different chromosomes [16].

Mice carrying homozygous deletions of the paralogous genes *Hoxa9, Hoxb9*, and *Hoxd9* demonstrated compromised alveolar morphogenesis and secretory differentiation [19]. Defects were characterized as alveolar hypoplasia, with gland morphology after parturition resembling that of a mid-pregnant animal. Single mutant lines disrupted for these three genes showed no defects. However, homozygous *Hoxd9* disruption did show a reduction in pup survival, but only in the context of *Hoxa9* and *Hoxb9* heterozygosity. Double mutant combinations showed genetic interactions suggesting cooperative function. The tissue compartment in which these genes function was not determined. More recently, disruption of *Hoxa5*, was shown to lead to precocious alveolar development but impaired lactation, with function being required in the stroma [20].

In this paper we report the phenotypic, gene expression, and transplantation analysis of mammary glands from female mice carrying a targeted disruption mutation of the *Hoxd10* gene, as well as from mice carrying simultaneous disruptions of both *Hoxd9* and *Hoxd10* [21]. Despite developmentally regulated expression in the mammary gland, we demonstrate a primarily systemic function for *Hoxd10* that is required for alveolar differentiation and secretory activation in the epithelial compartment during lactation, as well as a genetic interaction between *Hoxd9* and *Hoxd10* that increases the severity of alveolar defects.

## Results

### Disruption of *Hoxd10* Leads to Impaired Lactation as a Single Gene Mutation, and this Effect Is Enhanced by Simultaneous Disruption of *Hoxd9*

In preliminary phenotypic analysis of the Δ*Hoxd10* and *ΔHoxd9/d10* alleles [21, 22], a noticeable percentage of litters born to homozygous mothers of either line appeared dehydrated or failed to survive. Pups generally died within the first few days, with little or no milk in their stomachs.

To quantify the impact of the homozygous mutant phenotypes on pup survival, we examined homozygous, heterozygous, and wild type littermates from the *ΔHoxd10* and *ΔHoxd9/d10* lines for their ability to support litters through their first lactation as a measure of lactational fitness. Table 1 shows the frequency of failure to maintain a litter as a function of maternal genotype in the *ΔHoxd10* line. In this set of crosses, no wild type mice (0/14) failed to support litters and 18% (6/33) of *ΔHoxd10* heterozygotes failed to support litters (*p* = 0.159). In contrast, 40% (10/25) of *ΔHoxd10* homozygotes failed to maintain their first litter (*p* = 0.007 v WT; *p* = 0.066 v heterozygote).

**Table 1:** Frequency of failure to maintain a litter as a function of maternal genotype

Genotype	# Failed (%)	*p* value
Hoxd 10 ^+/+^	0/14 (0%)	
Hoxd 10 ^+/−^	6/33 (18%)	0.15g^a^
Hoxd 10 ^−/−^	10/25 (40%)	0.007^a^; 0.066^b^
Hoxd 10 ^+/+^	2/12 (17%)	
Hoxd 10 ^+/−^	4/15 (27%)	0.066^a^
Hoxd 10 ^−/−^	10/16 (63%)	0.015^a^; 0.045^b^

^a^Compared to WT based on chi-square test for two proportions

^b^Compared to Heterozygotes based on chi-square test for two proportions

Table 1 also shows the frequency of failure to maintain a litter as a function of maternal genotype for the *ΔHoxd9/d10* line. In this set of crosses, 17% (2/12) of wild type dams failed to support litters. Similarly, 27% (4/15) of heterozygous *ΔHoxd9/d10* did not maintain litters (*p* = 0.66). In contrast, 63% (10/16) of homozygous *ΔHoxd9/d10* dams failed to support litters (*p* = 0.015 v WT; *p* = 0.045 v heterozygote).

In order to quantify the effect of impaired lactation on pup nutrition, to ensure that pups born to homozygous Δ*Hoxd10* dams were able to gain weight normally, and to confirm that the lactation phenotype was not due to failure of pups to suckle properly, we conducted a cross-fostering experiment in which age-matched litters were standardized for pup number (*n* = 6) and exchanged between dams of the Δ*Hoxd10* line and wild type (CD1) control dams at approximately 24 h postpartum (L1). Thus, dams of the Δ*Hoxd10* line always nursed healthy, CD1 pups, while CD1 dams always nursed pups from the Δ*Hoxd10* line that had been fed by either wild type, heterozygous, or homozygous dams for the first 24 h.

**Fig. 1:**
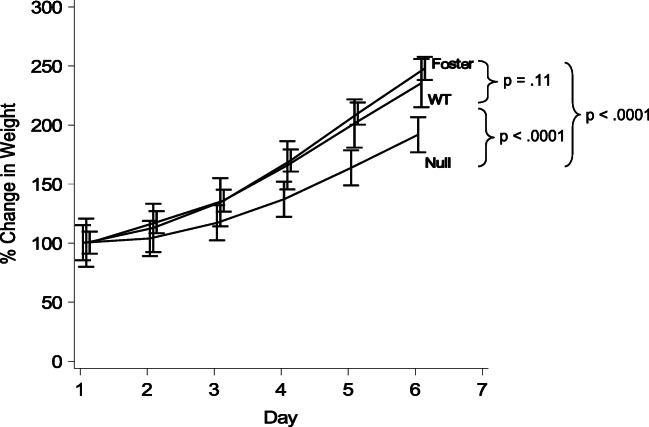
Disruption of *Hoxd10* leads to a reduction in pup survival. Cross-foster analysis of lactational fitness of homozygous *ΔHoxd10* dams: normalized percent weight gain per pup over time as a function of foster mother genotype. Bars represent 95% confidence intervals

Figure 1 shows the normalized percent weight gain per pup as a function of foster mother genotype assayed through the first six days of lactation. Δ*Hoxd10* pups nursed by CD1 foster mothers thrived regardless of the genotype of the mother to which they were born and gained an average of 155% of their birth weight. Similarly, wild type pups nursed by wild type controls from the Δ*Hoxd10* line also thrived, gaining an average of 135% of their birth weight. These two growth curves were not statistically different from one another (*p* = 0.11). In contrast, wild type pups fostered to homozygous *ΔHoxd10* dams gained only 91% of their birth weight, on average. This growth curve was statistically different from the curves of both the CD1 foster mothers and wild type foster mothers from the Δ*Hoxd10* line (*p* < 0.0001). These data indicated that the loss of *Hoxd10* in the nursing dam resulted in the inability of the dam to provide sufficient milk to sustain a litter properly.

### Disruption of *Hoxd10* and *Hoxd9/d10* Results in Morphological and Histological Defects in the Mammary Gland during Lactation

To determine which aspect of mammary gland development or functional differentiation was affected in homozygous Δ*Hoxd10,* and whether these phenotypes might be augmented in *ΔHoxd9/d10* mice, we examined stained whole mount preparations of mammary glands from homozygous and wild type mice at key phases of development.

**Fig. 2:**
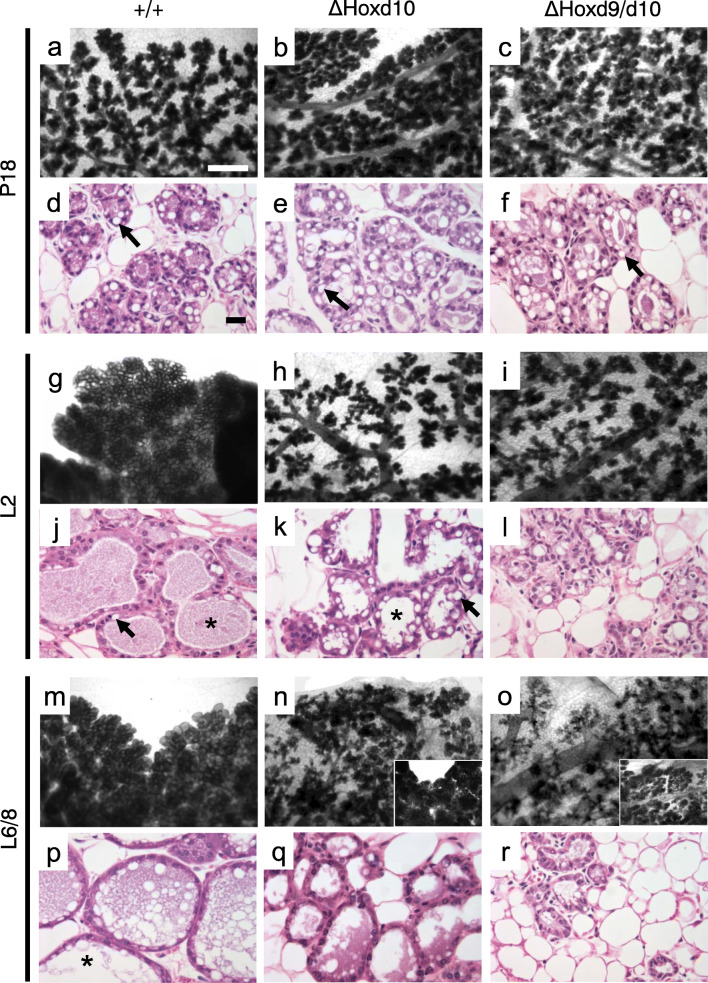
Whole mount preparations and histological analysis of mammary glands during pregnancy and lactation. Genotype of the mouse from which the gland was derived is shown above the column to which it applies. Developmental phase is shown to the left of the row to which it applies. **a-c** Normal morphology. Alveoli present along ductal tree. Scale bar = 0.5 mm. **d-f** Normal histoarchitecture. Alveoli present and contain large cytoplasmic lipid droplets (arrow). Scale bar = 20 μm. **g** Normal morphology. Alveoli are expanded and minimal adipose tissue visible. **h**, **i** Abnormal morphology associated with failure to maintain a litter. Alveoli are not expanded and adipose tissue visible. **j** Normal histoarchitecture showing expanded lumina (asterisk), sparse adipose tissue and relative absence of large cytoplasmic lipid droplets. **k** Abnormal histoarchitecture showing condensed lumina, with occasional expanded lumina (asterisk), retention of adipose tissue, and presence of large cytoplasmic lipid droplets (arrow). **l** Abnormal histoarchitecture showing highly condensed lumina, retention of adipose tissue, and rare small cytoplasmic lipid droplets. **m** Normal morphology. **n** Abnormal morphology associated with failure to maintain a litter. Inset: normal morphology of a gland from a successfully lactating homozygote, lactation day 6. **o** Abnormal morphology associated with failure to maintain a litter. Inset: near-normal morphology of a gland from the same mouse, lactation day 8. **p** Normal histoarchitecture with expanded lumina (asterisk). **q** Abnormal histoarchitecture showing condensed lumina, retention of adipose tissue, and absence of cytoplasmic lipid droplets. **r** Abnormal histoarchitecture showing highly condensed lumina, retention of adipose tissue, and absence of cytoplasmic lipid droplets

Glands from wild type and homozygous *ΔHoxd10* and *ΔHoxd9/d10* mice harvested at virgin stages (not shown) or pregnancy day 18 (P18) appeared normal (Fig. 2a-c). All wild type glands harvested at lactation day two (L2) or six (L6) showed characteristic formation of alveolar clusters that had expanded during lactation (Fig. 2g and m). In contrast, homozygous *ΔHoxd10* and *ΔHoxd9/d10* dams that failed to maintain a litter showed gland morphology at L2 indistinguishable from that normally observed at P18 (Fig. 2h and i). Homozygous Δ*Hoxd10* and *ΔHoxd9/d10* dams that were able to support litters until L6/8 also showed glands with impaired lactation (Fig. 2n and o), but also showed regions or entire glands that had undergone lactogenesis with successfully expanded alveoli (Fig. 2n and o insets).

To assess whether the morphological changes observed in whole mount preparations reflected developmental arrest of alveoli in late pregnancy, we examined histological preparations representative of each phenotype at P18, L2, and L6/8. All mice exhibited histological characteristics indistinguishable from wild type glands at P18 (Fig. 2d-f). Alveolar lumina were condensed and large cytoplasmic lipid droplets were present in the majority of alveolar epithelial cells. At L2, glands of wild type mice showed normal histology (Fig. 2j) with expanded alveolar lumina and a characteristic decrease in the average size and number of cytoplasmic lipid droplets due to secretion into the lumen. In contrast, Δ*Hoxd10* mutants that failed to maintain litters showed a failure of alveoli to completely expand as well as the retention of large cytoplasmic lipid droplets characteristic of P18 (Fig. 2k). Homozygous *ΔHoxd9/d10* mutants typically showed small alveoli and a reduction in visible cytoplasmic lipid droplets (Fig. 2l). At L6, wild type glands showed normal histoarchitecture (Fig. 2p) while the impaired phenotype in affected *ΔHoxd10* glands could persist at least through L6 (Fig. 2q). The most severely affected glands in *ΔHoxd9/d10* mutants (L8) appeared to have begun involution with reduced alveolar structures and increased adipose stroma (Fig. 2r).

With respect to penetrance, during early lactation, 66% of homozygous *ΔHoxd10* mice and 94% of homozygous *ΔHoxd9/d10* mice showed altered whole mount morphology of one or more glands. Surprisingly, 34% of wild type control mice also showed altered whole mount morphology of one or more glands (Online Resource 1a). These observations suggested an underlying propensity of lactation impairment in the C57BL/6 parental line used, and loss of *Hoxd10* and, to a greater degree, *Hoxd9/d10* leads to a higher probability of lactation failure.

To evaluate the degree to which the impaired lactation phenotype was expressed as a function of genotype (expressivity), histological preparations were evaluated for the percentage of the gland affected for wild type versus homozygous mice. Glands of wild type mice showed that, while 57% of the glands were not affected, 7% of glands showed regions of abnormal morphology, and 36% of all glands evaluated showed complete failure. In contrast, homozygous *ΔHoxd10* mutants showed an average of 10% of glands with affected regions, and 63% showing complete failure. Finally, glands from homozygous *ΔHoxd9/d10* mice showed 10% with affected regions, but had the highest proportion of glands with complete failure at 74% (Online Resource 1b).

*Hoxd10* disruption is known to disrupt hindlimb, but not forelimb innervation [21]. To assess whether there was an effect of *Hoxd10* or *Hoxd9/d10* disruption on gland development as a function of anterior-to-posterior gland position, we examined whole mount preparations of glands #1 through #5 from one side of each homozygous animal harvested between L2 and L8 for evidence of lactation failure. We found no statistical difference in the probability that a specific gland position would be affected more than any other (Online Resource 1c).

### *Hoxd9* and *Hoxd10* Expression Are Coordinately Regulated during Mammary Gland Development

Given that simultaneous disruption of *Hoxd9* and *Hoxd10* led to more severely impaired lactation than *Hoxd10* disruption alone, and that *Hoxd9* and *Hoxd10* are known to interact functionally in nervous system development [22], we wished to determine whether *Hoxd9* and *Hoxd10* were coordinately expressed in the mammary gland.

**Fig. 3:**
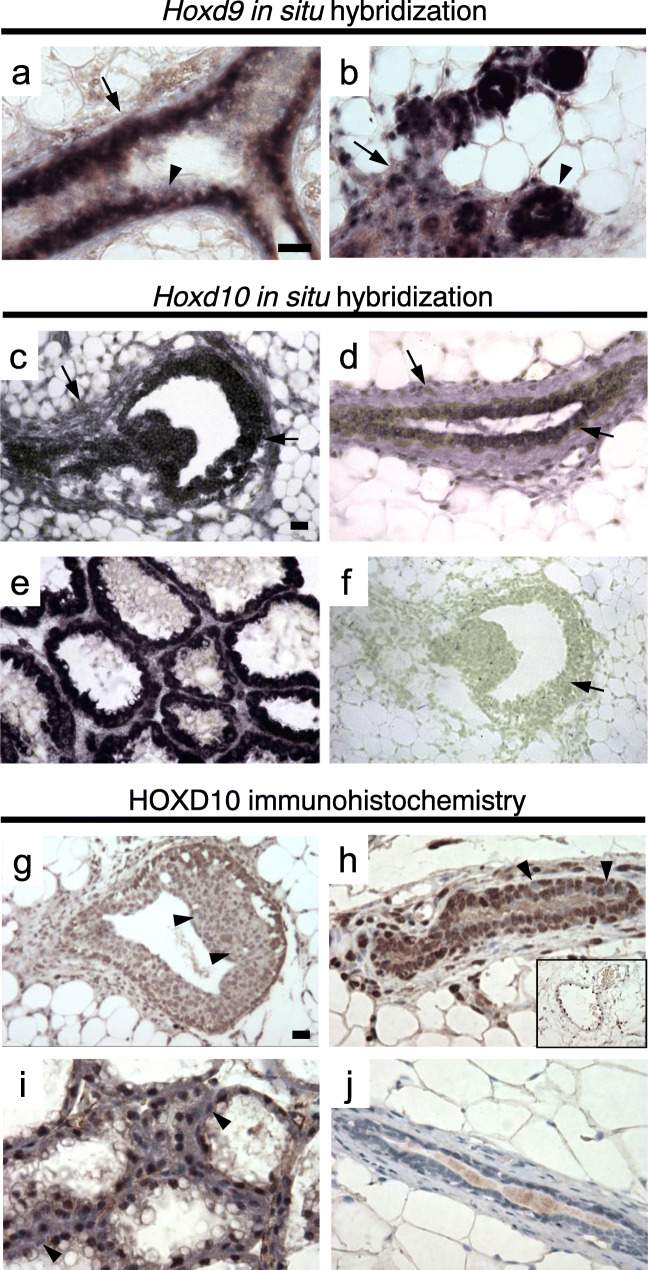
In situ hybridization for *Hoxd10 and Hoxd9,* and immunohistochemistry for HOXD10*.***a** In situ hybridization for *Hoxd9*. Duct of a mature virgin mouse. Hybridization was observed primarily in luminal epithelial cells (small arrow) and a minority of periductal stromal cells (large arrow). Scale bar = 20 μm. **b** Developing alveoli in mid-pregnancy. Hybridization was observed in all epithelial cells of the alveoli (small arrows). Expression was also detected in periductal and perialveolar stroma (large arrow). The pattern observed in ducts of virgin mice was largely preserved in ductal structures during pregnancy (not shown). Sense strand controls showed no hybridization (data not shown). **c** Developmental in situ hybridization for *Hoxd10*. Terminal end bud in a 5-week-old female. Epithelium of the end bud (both body cells and cap cells) are stained positively with the blue-black precipitate (arrow), as is the condensing periductal stroma (arrowhead). Scale bar = 20 μm. **d** Mature duct in a 10-week-old female. Expression in both the epithelium (arrow) and the periductal stroma (arrowhead) is maintained. **e** Lactation day 10. Expanded alveoli stained strongly. **f** Terminal end bud in a 5-week-old female. Sense strand negative control showing no epithelial or stromal staining (arrow). **g** Immunohistochemical analysis of HOXD10 protein expression. Terminal end bud in a 5-week-old female. Epithelium of the end bud (both body and cap cells) are stained positively, with some body cells showing notably lower expression (arrows). Scale bar = 20 μm. **h** Mature duct in a 10-week-old female. Expression in both the epithelium and the periductal stroma is maintained. Rare individual cells do not stain for HOXD10 protein (arrows). HOXD10 expression is also prominent in vascular endothelial cells (inset). **i** Lactation day 10. Prominent nuclear staining in virtually all alveolar cells. Rare epithelial cells contacting the lumen show no staining (arrows). **j** Mature duct in a 10-week-old homozygous *ΔHoxd10* female showing no staining demonstrating specificity of the antibody

By in situ hybridization, *Hoxd9* was expressed primarily in luminal epithelium and in a minority of periductal stromal cells in virgin ducts (Fig. 3a). Consistent with its demonstrated role in alveolar development, *Hoxd9* was well-expressed in the epithelium of alveolar structures during pregnancy (Fig. 3b) as well as in isolated periductal stromal cells. *Hoxd10* also showed expression in both the epithelium and stroma during development. In 5-week-old mice, *Hoxd10* expression was concentrated in body cells of terminal end buds and cap cells (Fig. 3c). Expression was also observed in the condensed periductal stroma. This expression pattern was maintained in mature glands at 10 weeks of age, which also showed expression in myoepithelial and luminal epithelial cells (Fig. 3d). Qualitatively, *Hoxd10* mRNA expression appeared to be elevated in lactation (Fig. 3e), as judged by more rapid and intense accumulation of blue-black precipitate. Sense strand control hybridizations showed no staining (Fig. 3f).

To determine whether the spatial and temporal pattern of *Hoxd10* mRNA expression was recapitulated at the protein level, we performed immunohistochemical detection of HOXD10 protein. Nuclear HOXD10 was detected in a majority of cells in the mammary gland, including body cells and cap cells of the terminal end bud (Fig. 3g), luminal epithelium of differentiated ducts (Fig. 3h), periductal stromal cells and other isolated stromal cells (Fig. 3g and h), and vascular endothelium (Fig. 3h inset). HOXD10 expression was also detected during pregnancy (data not shown) and lactation (Fig. 3i). Rare HOXD10 negative cells could also be observed in all tissue compartments at these phases (arrows). Control tissue from *ΔHoxd10* homozygotes showed no staining, confirming antibody specificity (Fig. 3j).

Finally, we performed qPCR on mammary tissue at lactation day two to evaluate relative gene expression of *Hoxd9* and *Hoxd10*, as well as other paralagous Hox genes which could potentially play a compensatory role in the absence of *Hoxd9* or *Hoxd10*. As expected, *Hoxd10* expression was completely ablated in *ΔHoxd10* and *ΔHoxd9/d10* homozygous mice, and *Hoxd9* expression was absent in *ΔHoxd9/d10* homozygous mice (Online Resource 2a). However, expression of *Hoxd9* was reduced by more than half in *ΔHoxd10* homozygous mice relative to wild type controls, indicating that the loss of *Hoxd10* expression results in the down regulation of *Hoxd9*. These data are consistent with those seen in the lumbar spinal cord of developing mice where *Hoxd10* and *Hoxd9* are coordinately expressed in the same regions and loss of *Hoxd10* results in the down regulation of *Hoxd9* [23]. When we analyzed the gene expression of other paralogous Hox genes, we found no significant differences in gene expression except for *Hoxb9*, which showed a relatively higher expression in *ΔHoxd9/d10* glands than *ΔHoxd10* or wild type (Online Resource 2b).

### Mammary Glands of *ΔHoxd10* and *ΔHoxd9/d10* Mutants Show Reduced Expression and Activation of Key Lactation Associated Proteins

To investigate mechanism(s) by which glands from *ΔHoxd10* and *ΔHoxd9/d10* homozygotes failed to undergo alveolar expansion and secretion, we evaluated expression of several proteins and genes known to be required for, or associated with, lactation.

**Fig. 4:**
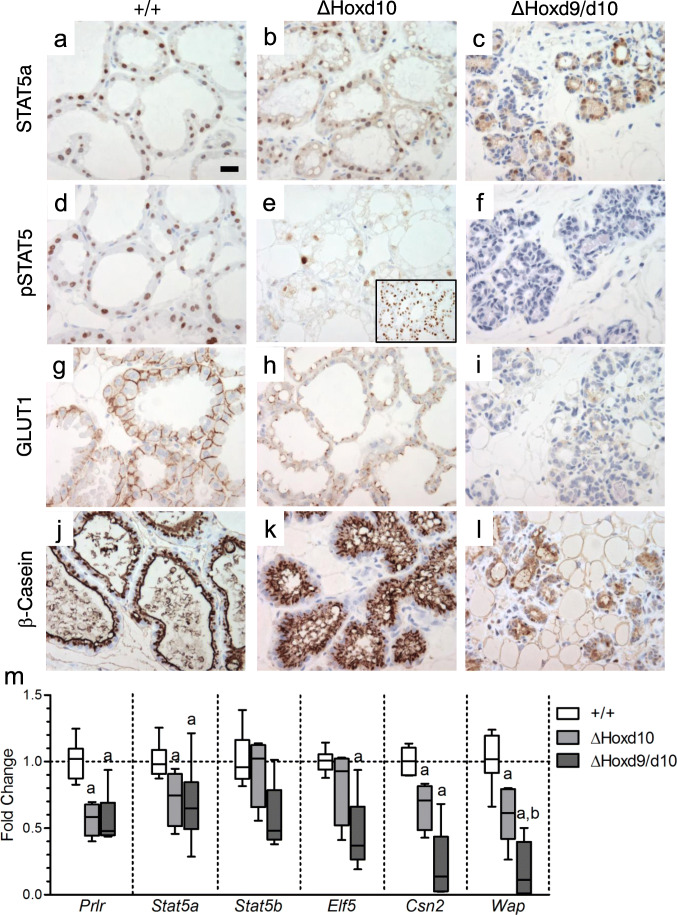
STAT5a, phosphorylated STAT5, GLUT1, and β-Casein expression and localization by immunohistochemistry. Genotype of the mouse from which the gland was derived is shown above the column to which it applies. Antibody used is shown to the left of the row to which it applies. All mammary glands shown were harvested at lactation day 2. **a** STAT5a is expressed and localized primarily in the nucleus. Scale bar = 20 μm. **b** STAT5a is expressed and localized in the cytoplasm and nucleus. **c** STAT5a expression is attenuated in severely affected alveoli. **d** STAT5 is phosphorylated and localized primarily in the nucleus. **e** Phosphorylation of STAT5 is reduced in failed regions. However, STAT5 is phosphorylated and localized primarily in the nucleus in morphologically normal regions (**f**) Absence of STAT5 phosphorylation (**g**) GLUT1 is expressed and localized primarily in the basolateral membrane of alveolar cells. **h** GLUT1 expression is reduced and localized primarily in the cytoplasm. **i** GLUT1 expression is dramatically reduced. **j** β-Casein is expressed and localized primarily in the expanded lumina. **k** β-Casein is expressed, but localized primarily in the cytoplasm of alveolar cells. **l** β-Casein is expressed in some alveolar cells, with dramatically reduced levels in severely affected alveoli. **m** QPCR analysis of lactation associated genes in the mouse mammary gland at lactation day 2. a: versus WT: *p* ≤ 0.05; b: versus *ΔHoxd10*: *p* ≤ 0.05; whiskers represent range

Prolactin receptor (PRLR), Janus kinase 2 (JAK2), and signal transducer and activator of transcription 5 (STAT5) belong to a signaling pathway required for alveologenesis, proper milk protein gene expression, and continued maintenance of differentiated alveolar cells. Knockout studies have revealed that the loss of *Prlr, Jak2, or Stat5a* each result in a lack of alveolar proliferation and differentiation [24–27]. While STAT5a showed an expected pattern of nuclear staining in both wild-type and *ΔHoxd10* mice at L2 (Fig. 4a and b, respectively), *ΔHoxd9/d10* mice showed decreased STAT5a intensity in most expressing cells, with a complete absence in a majority of cells (Fig. 4c). Phosphorylation of total STAT5 (Y694) in wild type glands showed a strong staining pattern within the nucleus which coincides with the expression pattern of STAT5a seen in wild type glands (Fig. 4d). In failed regions of *ΔHoxd10* glands we saw reduced intensity of pSTAT5 and a reduction in the number of cells positive for pSTAT5 (Fig. 4e). However, pSTAT5 in unaffected glands from lactating *ΔHoxd10* mice are similar to those seen in wild type mice (Fig. 4e inset). In *ΔHoxd9/d10* glands, there is a reduction in pSTAT5 intensity, as well as a decrease in the total number of pSTAT5 positive cells when compared to *ΔHoxd10* glands (Fig. 4f).

Glucose transporter, GLUT1, is normally localized to the basolateral membrane where it supports the increased demand for glucose for synthesis of lactose, lipids, and milk proteins. The rate of glucose uptake by mammary epithelial cells was shown to be the determining factor in the rate of milk production [28]. In wild type glands we observed the expected prominent basolateral expression of GLUT1 in alveolar cells (Fig. 4g). However, *ΔHoxd10* glands showed a reduction in expression and generalized failure to localize GLUT1 protein (Fig. 4h) while *ΔHoxd9/d10* glands showed very little GLUT1 expression at all (Fig. 4i). Since GLUT1 expression is dependent on prolactin levels during lactation, the reduction of GLUT1 within the mutant mammary glands is consistent with disrupted prolactin signaling.

Consistent with reduced STAT5 phosphorylation, expression and localization of the milk protein β-casein was also altered. β-casein was readily detected in both wild type (Fig. 4j) and *ΔHoxd10* tissue (Fig. 4k) suggesting no obvious defect in milk protein synthesis. However, β-casein was located primarily in the apical cell surface in cells of wild type mice, whereas the protein was localized primarily to the cytoplasm of alveolar cells in *ΔHoxd10* homozygotes. In contrast, severely affected regions of *ΔHoxd9/d10* homozygotes showed a dramatic decrease in β-casein staining, as well as apparent retention in the cytoplasm (Fig. 4l) suggesting a disruption in both synthesis and secretion.

With respect to the prolactin signaling cascade at the RNA level, quantitative PCR of *ΔHoxd10* and *ΔHoxd9/d10* homozygous glands during early lactation revealed an approximate 2-fold reduction in *Prlr* mRNA expression in comparison to wild-type controls (Fig. 4m). Further, mRNA expression of *Stat5a* was significantly reduced by approximately 1.4-fold in both knockout lines, while *Stat5b* was not significantly different between knockout lines and wild-type. *Stat5b* is not required for alveolar development and lactation, but may be able to partially compensate for the loss of *Stat5a* [29, 30]. *Jak2* mRNA expression, which is required for activation of *Stat5*, remained unaltered in both knockout lines relative to wild-type controls (data not shown).

Looking at downstream effectors of prolactin signaling, we also analyzed mRNA expression of E74-like factor 5 (*Elf5*), a transcription factor regulated by prolactin that is essential for alveolar development and lactation [31]. The Stat5a promoter contains a conserved *Elf*-binding site, and the loss of *Elf5* is associated with a reduction in *Stat5a* expression and attenuated STAT5 activity [32]. We did not find a significant reduction in mRNA expression of *Elf5* in *ΔHoxd10* homozygotes; however, *ΔHoxd9/d10* homozygotes showed a 2.3-fold reduction relative to wild-type controls.

Expression of β-casein (*Csn2*), whose promoter contains STAT5 binding sites and is regulated by prolactin, insulin, and hydrocortisone, is dramatically elevated after parturition [33, 34]. Consistent with our immunostaining results in *ΔHoxd10* homozygotes, we found that mRNA levels of β-casein during lactation were 1.5-fold lower relative to wild type controls, and in *ΔHoxd9/d10* homozygotes it was reduced by 4.7-fold. We also evaluated expression of whey acidic protein (*Wap*), which is highly induced by STAT5 during lactation and can be trans-activated by ELF5 [24, 32, 35]. We found expression levels of *Wap* were reduced by 1.7-fold in *ΔHoxd10* homozygotes and 5.5-fold in *ΔHoxd9/d10* homozygotes. These data indicate that downstream targets of the prolactin signaling pathway are also affected by a loss of *Hoxd10* and, to a greater degree, by the additional loss of *Hoxd9*.

### Homozygous *ΔHoxd10* and *ΔHoxd9/d10* Mutants Show Increased Expression and Activation of STAT3, Altered Cell State, and Recruitment of CD45+ Immune Cells

**Fig. 5:**
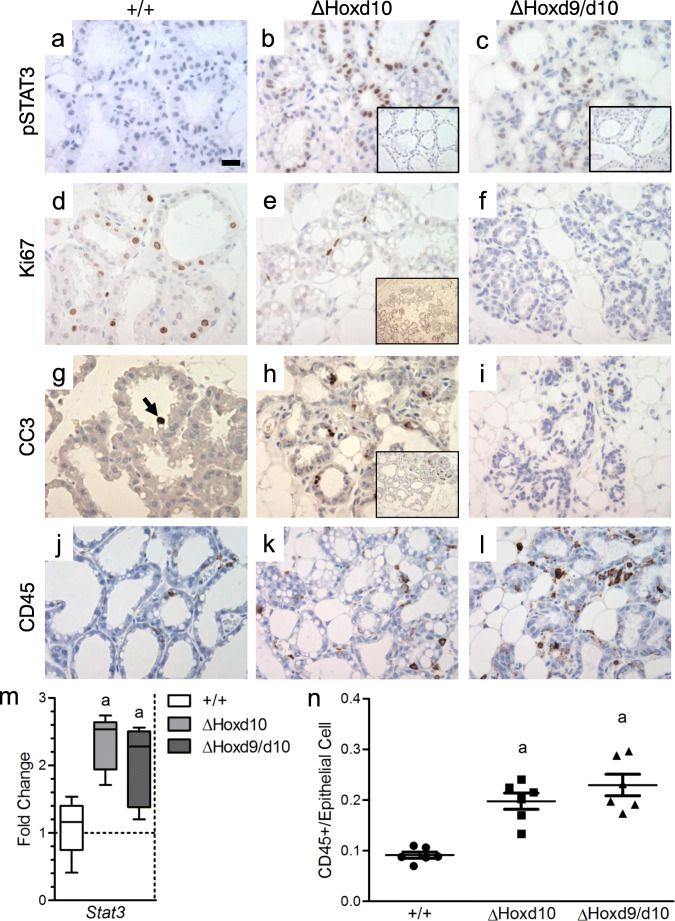
Phosphorylated STAT3 (pSTAT3), Ki67, cleaved caspase-3 (CC3), and CD45 expression and localization by immunohistochemistry. Genotype of the mouse from which the gland was derived is shown above the column to which it applies. Antibody used is shown to the left of the row to which it applies. All mammary glands shown were harvested at lactation day 2. **a** Phosphorylation of STAT3 is undetectable. Scale bar = 20 μm. **b** Phosphorylation of STAT3 is found in the nuclei of affected regions. Absence of pSTAT3 is seen in unaffected regions (inset). **c** Phosphorylation of STAT3 is found in the nuclei of affected regions. Absence of pSTAT3 seen in unaffected regions (inset). **d** Prominent Ki67 expression in most nuclei. **e** Loss of Ki67 expression seen in affected regions. Ki67 expression clearly demarcates failed versus functional regions of the gland (inset). **f** Loss of Ki67 expression seen in affected regions. **g** Rare apoptotic cell expressing cleaved caspase-3(CC3) (arrow). **h** Increased apoptosis in affected region detected by cleaved caspase-3 expression. Increased apoptosis in affected region clearly demarcates failed versus functional regions of the gland (inset). **i** Severely affected regions rarely show apoptotic cells expressing cleaved caspase-3 (**j**) CD45+ cells are found sparsely populated throughout the stroma and epithelium. **k** CD45+ cells are found abundantly throughout the stroma and epithelium. **l** CD45+ cells are found abundantly throughout the stroma and epithelium. **m** QPCR analysis of *Stat3* in the mouse mammary gland at lactation day 2. a: versus WT: *p* ≤ 0.05; whiskers represent range. **n** Quantification of the number of CD45+ cells per epithelial cell. There is a significant increase in the number of CD45+ cells present in the mammary gland for *ΔHoxd10* and *ΔHoxd9/d10* homozygotes at L2. a: versus WT: *p* ≤ 0.05; error bars represent mean ± S.E.M.

To determine if there were any changes within the mammary gland that would indicate a shift towards involution, we looked at the phosphorylation status of STAT3, cell state by Ki67 staining, and the presence of immune cells two days after parturition. Signal transducer and activator of transcription 3 (STAT3) is known to be activated by phosphorylation at the start of involution and is reciprocal in expression pattern and function to STAT5. Previous knockout studies revealed that a loss of STAT3 led to a failure of the lactating mammary gland to undergo apoptosis and initiate the first phase of involution [36, 37]. As expected, with wild-type glands we did not see activation of STAT3 in alveolar cells during lactation (Fig. 5a). However, we did observe an inappropriate activation of STAT3 in *ΔHoxd10* and *ΔHoxd9/d10* homozygote alveolar cells (Fig. 5b and c). Again, there were areas within mutant glands that appeared to function normally and did not show activation of STAT3, consistent with patterns in lactating wild-type glands (Fig. 5b and c insets). In *ΔHoxd10* and *ΔHoxd9/d10* homozygotes, quantitative PCR of mammary glands at L2 show a 2-fold increase in the expression of *Stat3* relative to wild-type controls (Fig. 5m). The decrease in expression of lactogenic signaling factors and downstream targets in conjunction with an increase in *Stat3* expression and activation, suggest that the mammary glands in *ΔHoxd10* and *ΔHoxd9/d10* homozygous mice have not only failed to initiate lactogenesis, but have undergone involution as well.

Since the activation of STAT3 in *ΔHoxd10* and *ΔHoxd9/d10* homozygotes suggested premature involution, we evaluated cell cycling state via Ki67 expression, as well as apoptosis via cleaved caspase-3 expression. Pronounced differences between functional and impaired regions were observed. Staining for Ki67 was observed in approximately 80–90% of secretory epithelium in wild type tissues and unaffected regions of *ΔHoxd10* and *ΔHoxd9/d10* tissue (Fig. 5d). In contrast, affected regions of *ΔHoxd10* and *ΔHoxd9/d10* glands showed relatively few Ki67 positive cells (<20%) (Fig. 5e and f). In glands where regional impairment was observed, Ki67 loss clearly demarcated the boundary of the impaired region (Fig. 5e inset).

With respect to apoptosis, in wild type tissues, unaffected regions of *ΔHoxd10* tissues*,* and *ΔHoxd9/d10* tissues, cleaved caspase-3 staining showed only rare positive cells (<<1%) (Fig. 5g, h inset, and i). In contrast, affected regions of *ΔHoxd10* glands showed pronounced expression of cleaved caspase-3 (Fig. 5h). These data suggest that failure to undergo secretory differentiation in *ΔHoxd10* homozygotes leads to cell cycle exit and induction of apoptosis. However, *Hoxd9/d10* loss leads to cell cycle exit with what appears to be primarily developmental arrest.

During normal involution there is an early increase in neutrophils followed by an increase in macrophages and other lymphocytes that facilitate the removal of apoptotic cells and residual milk [38]. Consistent with the premature induction of involution, we observed an increase in the number of lymphocytes (CD45+) present within the glands of *ΔHoxd10* and *ΔHoxd9/d10* homozygous mice relative to wild type mice (Fig. 5j-l). We found that there was a 2-fold increase in the number of CD45 positive cells per epithelial cell in mutant glands compared to wild-type controls (*p* = 0.0001, Fig. 5n). These data, along with the increase of expression and activation of STAT3, and the disruption of the cell cycle indicate that *ΔHoxd10* and *ΔHoxd9/d10* mutant mammary glands two days after parturition are undergoing premature involution.

### Serum Levels of Estradiol, Progesterone, and Prolactin Are Not Altered, and Pituitary Isografts Do Not Rescue the *Hoxd10* Mutant Phenotype

The regional nature of lactation failure argued against a change in overall systemic hormone levels required for lactation. However, to determine more definitively if a disruption in serum hormone levels might contribute to the lactation phenotype observed in *ΔHoxd10* and *ΔHoxd9/d10* homozygotes, we evaluated the levels of three hormones, estradiol, progesterone, and prolactin, in early lactation. We found no statistical difference between wild-type controls and *ΔHoxd10* and *ΔHoxd9/d10* homozygotes at L2 (Online Resource 3a-c).

Despite the finding that baseline serum prolactin levels were unaltered in *ΔHoxd10* mice and that most animals possessed some mammary glands, or regions of function, we performed a pituitary isograft to ascertain whether increased prolactin would be sufficient to activate STAT5 in alveolar cells and consequentially improve lactogenesis. We harvested pituitaries from wild-type controls, transplanted them into the kidney capsule of pregnant *ΔHoxd10* homozygous mice (vs sham operated controls), and then harvested the mice two days after parturition. As expected, sham controls showed an overall failure to undergo proper lactogenesis, and there was no noticeable increase in pSTAT5 (Online Resource 3d). In *ΔHoxd10* homozygous mice that received a pituitary isograft, there was a slight increase in intensity of pSTAT5 by immunohistochemistry in most glands relative to sham controls (Online Resource 3e) with some glands showing regional high pSTAT5 consistent with observations in the intact animals (Online Resource 3e, inset). However, morphological and histological defects seen in earlier analyses corresponding to a loss of differentiated lactation remained in most glands. These data indicate that hormone levels of *ΔHoxd10* and *ΔHoxd9/d10* homozygotes are within normal physiological levels, are likely not contributing to the observed phenotype during lactation, and that an increase in prolactin is not sufficient to rescue the loss of differentiation during lactation.

### Reciprocal Transplantation Indicates that Impaired Lactation in *ΔHoxd10* Homozygotes Is Due Largely to a Systemic Role of *Hoxd10*

**Fig. 6:**
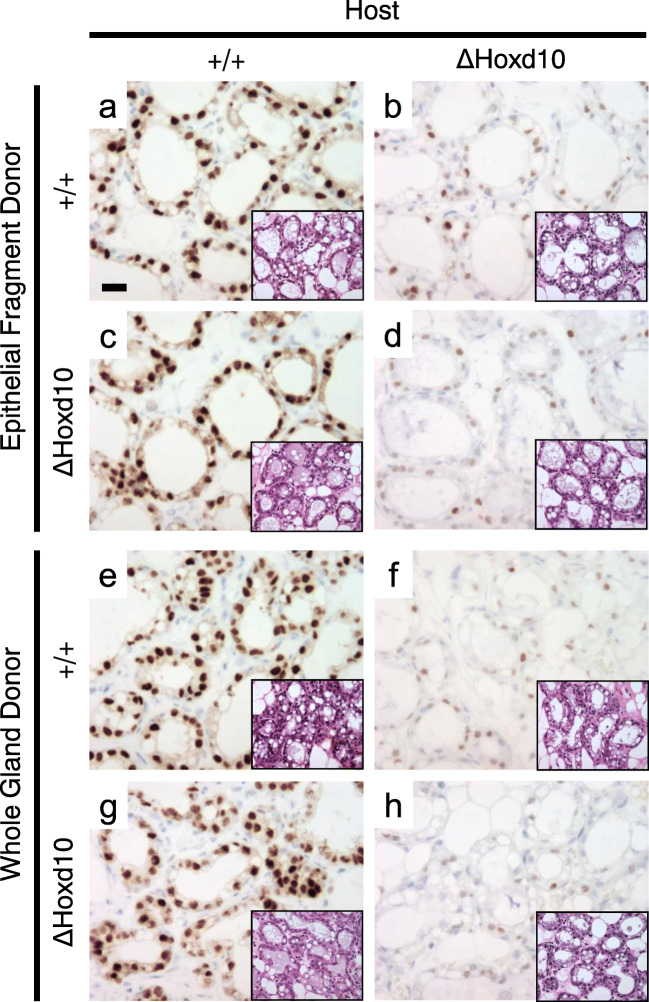
Immunohistochemical analysis of phosphorylated STAT5 expression of reciprocal epithelial fragment and whole gland transplantation. Host genotype is shown at the top of the column to which it applies. Tissue transplanted and donor genotype are shown to the left of the row to which it applies. Insets show corresponding H&E staining. Scale bar = 20 μm

To determine whether the lactation phenotype in *ΔHoxd10* homozygotes was indeed due to an epithelium-limited function, a mammary stroma-limited function, or whether it may be due to a systemic defect, we conducted two types of transplantation experiments, reciprocal epithelial fragment transplantation into epithelium-free fat pads, as well as reciprocal whole gland transplantation (Fig. 6). In reciprocal epithelial fragment transplantation, control wild type epithelium appeared normal when placed in a wild type host epithelium-free fat pad, as anticipated (Fig. 6a inset), with the expected phosphorylation of STAT5 (Fig. 6a). However, when wild type epithelium was placed in a *ΔHoxd10* mutant fat pad, phosphorylation of STAT5 was severely attenuated (Fig. 6b). When homozygous *ΔHoxd10* epithelium was placed in a wild type fat pad, glands were unexpectedly phenotypically normal (Fig. 6c inset) and showed STAT5 phosphorylation comparable to wild type/wild type controls (Fig. 6c vs a). As expected, control mutant epithelium into mutant fat pad showed defects in pSTAT5 that were indistinguishable from wild type epithelium into mutant fat pad (Fig. 6d vs b). These results indicate a prominent stromal or systemic function for *Hoxd10* that is able to confer a mutant phenotype on both wild type and *ΔHoxd10* epithelium.

To delineate between a mammary stromal vs. systemic function, we conducted whole mammary gland reciprocal transplantation. Results were identical with the results of the epithelial fragment transplantation when evaluated 12 h after parturition (Fig. 6e-h) with *Hoxd10* glands rescued in the context of a wild type environment, and the mutant phenotype conferred on wild type mammary glands in the context of a mutant systemic environment. These data indicate that the mutant phenotype is conferred upon wild type and *ΔHoxd10* epithelium via a systemic, not stromal, effect in the *ΔHoxd10* homozygous mouse.

## Discussion

In this paper we demonstrate that while *Hoxd9* and *Hoxd10* are expressed in a developmentally regulated manner in multiple cell types including mammary epithelial cells, as well as in the surrounding stromal cells during multiple stages of mammary gland development, *Hoxd10* functions largely systemically to confer defects in secretory activation at lactation.

Disruption of *Hoxd10* or co-disruption of *Hoxd9 and Hoxd10* failed to show any overt alterations to mammary morphology or histology during virgin development or pregnancy indicating these genes may not play a critical role in the mammary gland at these stages. However, with parturition, we demonstrated that the loss of *Hoxd10* alone is sufficient to disrupt alveolar expansion and secretion in 66% of nursing dams resulting in significant pup mortality, and, in many cases, inadequate nutrition to support normal growth of surviving pups. In *ΔHoxd9/d10* homozygous mothers, this phenotype was exacerbated, with almost all nursing dams having at least one gland that failed differentiation and lactation.

Previous work showed that loss of *Hoxd9* showed no effect on gland development as a single gene mutation, but did show function in combination with losses in other group 9 paralogs [19]. However, no transplantation experiments were done to demonstrate whether these defects were gland intrinsic or due to systemic alteration. In the current study, *Hoxd9* expression was downregulated over 2-fold during lactation in the *ΔHoxd10* homozygous dams, yet complete disruption of *Hoxd9* in conjunction with *Hoxd10* exacerbated the lactation defects observed in *Hoxd10* single mutants. This observation indicates that, in the absence of *Hoxd10*, *Hoxd9*, although significantly reduced, is still functionally sufficient such that single gene disruption of *Hoxd10* can maintain lactation in 34% of animals, but once *Hoxd9* is completely ablated only 6% of dams are able to maintain lactation. With the exception of a slight upregulation of *Hoxb9*, in *Hoxd9/d10* compound mutants, we saw no evidence to suggest an attempt at functional compensation by other group 9 or 10 paralogs. These observations highlight the importance of *Hoxd10* and *Hoxd9* as key co-regulators of alveolar differentiation and secretory activation.

Results from the hormone analysis show that estrogen and progesterone serum levels are consistently low during early lactation regardless of *Hoxd10* and *Hoxd9* disruption. Therefore, estrogen or progesterone are unlikely to inhibit lactation. Likewise, baseline serum prolactin levels are within the normal physiological range for early lactation to provide stimuli for milk production either regionally or on a gland-to-gland basis. That said, we did not measure suckling-induced PRL secretion which spikes transiently in response to suckling. It remains formally possible that there is a PRL secretion defect in the *Hoxd9/d10* double mutant [39]. However, given that affected regions can be immediately adjacent to functional regions, an overall defect in PRL secretion is unlikely to be present.

Using reciprocal transplantation assays we demonstrate that *Hoxd10* functions largely systemically to disrupt lactation despite no overt change in hormone levels as a consequence of disruption. Both epithelial fragment reciprocal transplants, as well as whole mammary gland reciprocal transplants demonstrated clearly that the wild type systemic environment was capable of rescuing pSTAT5 expression in *ΔHoxd10* mutant tissue (Fig. 6) and that the mutant phenotype can be conferred on wild type epithelium or whole mammary glands in the *ΔHoxd10* systemic environment. Because transplanted epithelium begins to involute soon after parturition due to milk stasis, we were only able to observe mammary glands within 12 h after parturition. At this point mutant and wild type glands were morphologically indistinguishable. In contrast, phosphorylation of STAT5 was prominent in all transplants into wild type hosts regardless of genotype, but showed marked decrease in all transplanted material into a mutant host. These data indicate that the mammary gland of *Hoxd10* mutants is capable of luminal expansion and differentiation immediately after parturition, but that a systemic factor or cell type is impairing the ability of STAT5 to be phosphorylated. Prolonged prevention of STAT5 activation would be consistent with the precocious involution observed.

The regional nature of the lactation phenotype is inconsistent with a global systemic alteration, which should affect all epithelial cells in a given animal equally. Although it is possible that specific areas are susceptible to a local threshold effect manifest as a consequence of alterations of cell function at a distant site (e.g. pituitary, ovary, etc). Rather, the local/regional lactation failure phenotype is more consistent with regional differences in the presence or function of a mammary extrinsic cell type that enters the gland during development. Mammary extrinsic cell types include immune cells, blood vessels, and neurons that invade the mammary fat pad and are recruited to the developing mammary gland as it fills the fat pad.

Despite the demonstration of a clear systemic role for *Hoxd10* in secretory activation in lactation, there are at least three lines of evidence to suggest that the Δ*Hoxd10* and *ΔHoxd9/d10* lactation defects are also influenced by an intrinsic local defect in the affected regions of the mammary gland itself. First, *Hoxd10* is expressed in a developmentally-regulated fashion in both the mammary epithelium and periductal stroma consistent with a function in one or both of the tissue compartments. Second, in most cases, not all glands in a given animal are affected to the same degree. In several cases, most of the glands in the animal were functional to the extent that the female was capable of supporting a litter, yet two or more glands failed to secrete. While unlikely given the transplantation results, these observations are formally consistent with a differential gland-limited requirement for *Hox10* function. Finally, affected regions could be observed immediately adjacent to phenotypically normal regions within an individual gland, regions that were most likely exposed to similar systemic hormones and growth factors. It is possible that the observed affected mammary glands in wild-type dams may be a response to suboptimal milk removal or suckling frequency since the litter size was limited to 6 pups [40, 41]. Nevertheless, the proportion of Δ*Hoxd10* and Δ*Hoxd9/d10* dams with affected glands is significantly higher than their wild-type counterparts suggesting that these mutants would be more sensitive to reduced milk removal or suckling frequency.

Mechanistically, at the level of the epithelial compartment of affected ducts, disruption of *Hoxd10* or *Hoxd9/d10* significantly decreased expression of at least four genes/proteins essential for lactation, including *Stat5a/b, Glut1, Prlr*, and *Elf5*. Importantly, disruption of any one of these genes in mammary epithelium is sufficient to disrupt lactation, though none of these disruptions phenocopy *Hoxd10* or *Hoxd9/d10* loss as single gene mutations. Thus, the phenotype observed in the Hox mutants due to changes in the epithelial compartment is likely due to combinatorial effects on the entire prolactin signaling cascade.

In lactation, elevated levels of prolactin initiate and maintain lactation by binding to PRLR which causes receptor dimerization and activation of the JAK2 kinase [42, 43]. JAK2 phosphorylates STAT5 which then dimerizes and translocates to the nucleus and binds to a distal promoter region of the β-casein and *Wap* genes (among others) to induce transcriptional activation [44]. Although it was shown that loss of STAT5a alone had little effect on β-casein gene expression due to the compensatory effects of STAT5b, overexpression of STAT5a was associated with increased synthesis and secretion of β-casein and other milk proteins [24, 45, 46]. *Elf5* has also been shown to bind the promoter region of *Stat5a* and that disruption of *Elf5* leads to the inactivation of STAT5 and down-regulation of β-casein and *Wap* [31, 32]. In *ΔHoxd10* and *ΔHoxd9/d10* homozygotes, the failure of mammary epithelial cells to differentiate functionally during lactation is associated with decreased mRNA expression of both *Prlr* and *Stat5a*, as well as a significant decline in STAT5a phosphorylation. Inactivation of STAT5a is, in turn, consistent with decreased β-casein and *Wap* expression, as well as decreased GLUT1 expression. In *ΔHoxd9/d10* homozygotes, the greater decrease in *Wap* expression and pSTAT5 levels is consistent with the more complete attenuation of *Stat5b* and *Elf5* expression.

After weaning, the mammary gland undergoes the process of involution which involves programmed cell death, compression of alveoli, removal of unneeded secretory epithelial cells, recruitment of immune cells (primarily neutrophils and macrophages for cell clearance), and finally the remodeling of the ductal tree. During the first stage of involution, within 12 h of forced weaning, STAT5 is deactivated and STAT3 is activated, along with the activation of programmed cell death [47, 48]. In *ΔHoxd10* and *ΔHoxd9/d10* dams, areas within the mammary gland that have reduced activation of STAT5 two days after parturition also show an upregulation and phosphorylation of STAT3. This also coincides with loss of Ki67 staining, an increase in mammary epithelial cell apoptosis as evaluated by cleaved caspase-3, as well as an influx of CD45+ immune cells to the mammary stroma. Together with the morphological features, these data indicate that the loss of *Hoxd10* or *Hoxd9/d10* leads to precocious involution within 48 h of parturition.

Taken together, these results suggest a model in which one or more undescribed systemic cell type is required for full activation of prolactin receptor-mediated signaling in the epithelial compartment of the mammary gland. There are at least three “systemic” cell types/structures that are recruited to the gland during development that could mediate this defect, immune cells, blood vessels, and neurons. However, we cannot rule out the possibility that failure of secretory activation is due to a defect at a more distant location as was recently shown for *Ptch1* disruption in immune cells of the pituitary and ovary that appear to impinge on development of the gland [49–51]. Tissue specific and cell type specific disruption of *Hoxd10* in these other cell types at different time points in mammary gland development will be necessary to dissect local effects due to the differential function of these cells/structures vs. systemic functions in other organs that impinge on the mammary gland indirectly. These long-term genetic studies are beyond the scope of this initial investigation.

## Materials and Methods

### Animal Strains and Maintenance

Mice carrying a targeted disruption mutation of the *Hoxd10* gene was described previously [21]. The disruption allele contains a neomycin resistance gene inserted into the homeobox thereby preventing *Hoxd10* proteins from functioning as DNA-binding transcription factors. The disruption allele was introduced into a 129Sv inbred background originally and was maintained thereafter by serial backcrossing to C57BL/6. Mice carrying a targeted disruption mutation of both the *Hoxd9* and *Hoxd10* genes were described previously [22]. The disruption construct was introduced into a 129Sv inbred background originally and was maintained thereafter by serial backcrossing to C57BL/6. Experimental mice used in phenotypic analysis were generated by a backcross-intercross breeding strategy. All mice used as donors in transplantation experiments had been backcrossed to C57BL/6 for at least eight generations, and all phenotypes were maintained in this inbred (histocompatible) background.

Balb/C (used only for gene expression analysis) and C57BL/6 inbred mice were obtained from breeding colonies maintained in our laboratory. CD1 mice used in cross-fostering experiments were obtained from Charles River Laboratories.

All experiments involving animals were approved by our institutional animal use committee.

### Tissue Harvest and Processing

At each developmental phase examined, mammary glands 1–5 from the right side of the mouse were removed, fixed in ice-cold 4% paraformaldehyde for 3 h, and hematoxylin or neutral red stained as whole mount preparations as described previously [52, 53]. The #2 and #3 glands were removed from the contralateral side of the mouse, fixed in ice-cold paraformaldehyde for 3 h and processed for in situ hybridization and immunohistochemistry as described previously [54].

### In Situ Hybridization

Digoxigenin labeled riboprobes, both sense and antisense, were produced by in vitro transcription of linearized plasmid using SP6 and T7 RNA polymerases.

The *Hoxd10* probe was a 500 bp fragment of the *Hoxd10* cDNA located 3′ to the homeobox (also provided by Dr. Denis Duboule) [55]. This probe was the pGBH500 subclone of *Hoxd10* and recognizes both major transcripts of the *Hoxd10* gene. For *Hoxd10* expression analyses, tissues from both Balb/C and C57BL/6 mice were embedded in paraffin, sectioned at 7um, and hybridized as described previously [54]. The developmental phases examined in both backgrounds were 5- and 10-week postpartum virgin, early pregnancy (P6-P8), late pregnancy (P17–19), and lactation day 10 (L10).

The *Hoxd9* probe used was the same ~750 bp fragment of the *Hoxd9* cDNA used in the Northern blot analysis, and has been used previously for expression analyses [55]. For *Hoxd9*, 10-week-old virgin and early pregnancy (P6–8) time points were examined.

### Whole Mount Analysis

Hematoxylin or neutral red stained whole mount preparations of glands 1–5 from the right side of the animal were examined under a dissecting microscope, scored individually by eye for phenotypic alterations, and photographed as necessary [52, 53] Scoring of lactation samples was used to corroborate the percentage of animals expressing the impaired lactation phenotype (penetrance), to assess the degree to which glands of a given genotype were affected (expressivity), and to evaluate whether an anterior-posterior position-specific effect on the lactation phenotype existed. The percent area of gland affected was compared across genotypes and gland position using longitudinal data analysis to account for repeat measurements within the same mouse for gland position. Contrasts were generated to perform pairwise comparisons between WT and each of the *ΔHoxd10* and *ΔHoxd9/d10* mutant groups.

### Histological Analysis

For virgin phases of development, regions of interest were identified, excised from the whole mount preparation, and embedded in paraffin for histological analysis. For pregnancy and lactation phases, the entire gland was embedded in paraffin for use in quantitative analysis of expressivity. In all cases, serial sections were cut at 3 μm and stained with hematoxylin and eosin. Histological sections were examined by microscopy, scored, and photographed as necessary.

### Quantitative PCR

Mammary glands were removed at lactation day two and snap frozen in liquid nitrogen. Total RNA was extracted using RNeasy Mini Kit (Qiagen). 2 μg RNA was reverse-transcribed (Random Primers and M-MLV Reverse Transcriptase, Invitrogen) following the manufacturer’s protocol. TaqMan Gene Expression Assays were purchased from Applied Biosystems. The resulting cDNA was analyzed using an Applied Biosystems 7500-Fast thermocycler for TaqMan quantitative PCR (Q-PCR) using standard conditions. Product accumulation was evaluated using the comparative Ct method (ΔΔCt method), with 18 s rRNA as an endogenous control for normalization.

### Immunolocalization

For detection of Hoxd10 protein, polyclonal antibodies were raised in rabbits using the peptide sequence, SQVESPEAKGGLPEDR, and verified by Western blot (Covance). Two antisera, designated 1159 and 1160 (1:200 dilution), produced qualitatively similar results. Tissue sections (3 μm) were subjected to antigen retrieval under Tris-HCl buffer, 0.1 M, pH 9.0 by heating to 120 °C for 10′ in a pressure cooker. Primary antibodies used include: rabbit anti-mouse β-casein [56], Ki67 (MIB-1) monoclonal antibody (Dako), Cleaved caspase-3 rabbit polyclonal antibody (Cell Signaling Technology), STAT5a rabbit polyclonal antibody (Santa Cruz Biotechnology), pSTAT5 (Tyr694) rabbit monoclonal antibody (Cell Signaling Technology), pSTAT3 (Tyr705)(D3A7) Rabbit monoclonal antibody (Cell Signaling Technology), and GLUT1 rabbit polyclonal antibody (Alpha Diagnostic International). Secondary antibodies (DakoCytomation) were conjugated to horseradish peroxidase. Detections were performed using the Vectastain Elite system.

### Lactational Fitness - Support of Litters

For both the *ΔHoxd10* and *ΔHoxd9/d10* lines, female mice representing all possible genotypes between 10 and 16 weeks of age were assayed for their ability to support litters in their first lactation. Litter size was determined on the day of birth and monitored daily thereafter. Dams were also evaluated daily for display of appropriate maternal behaviors (nesting, pup gathering, crouching, nursing). Litters of dams that killed their pups and those failing to display maternal behaviors were rejected from the analysis. Frequency of failure to maintain a litter, and percent of pups surviving as a function of maternal genotype were used as measures of lactational fitness.

Pairwise comparisons between maternal genotype of the proportion of dams who failed to maintain a litter were performed using the Fisher’s exact test. The chi-square test for trend was used to determine trends in failure rates across homozygous, heterozygous and wild type genotypes. Percent of pups surviving relative to the total number of pups born for each dam in each genotype was also calculated. The Wilcoxon rank-sum test was used to compare percent of pups surviving between maternal genotypes.

### Lactational Fitness - Comparative Assay of Litter Weight Gain

A cross-fostering experiment was performed to quantify the effect of impaired lactation on pup nutrition, to ensure that pups born to homozygous Δ*Hoxd10* dams were able to gain weight normally, and to establish that the lactation phenotype was not due to failure of pups to suckle properly. An analogous study was performed using the *ΔHoxd9/d10* line, but without cross-fostering.

For each study, litters were standardized to 6 pups on the day of birth and cross-fostered approximately 24 h postpartum (lactation day 1) with age-matched litters born to CD1 dams. Litters were counted and weighed daily between L1 and L6. To minimize the possibility that failure of pups to gain weight could be due to aberrant maternal behavior, only those litters having 5 or more pups that survived through L6 were used for quantitative analysis.

To correct for differences in starting weights of each litter, daily measurements of litter weight were normalized to litter weight at day 1. Specifically, the percent weight gain of each litter compared to day 1 was calculated for each dam in each genotype. The repeatedly-measured normalized percent weight gain were analyzed using growth curve models to compare the slope of litter weight gain between maternal genotypes.

### Epithelial Fragment and Whole Mammary Gland Transplantation

To test whether the lactation phenotype was intrinsic to the epithelium, we performed reciprocal tissue recombination transplantation in which wild type or mutant epithelium was transplanted contralaterally into the cleared fat pads of either wild type or mutant hosts in all four possible combinations. Transplanted glands and host controls were harvested on L1 within 12 h of parturition.

We also performed reciprocal whole mammary gland transplantation in which whole mammary glands from 3 week old wild type or homozygous *ΔHoxd10* donor mice were transplanted to the contralateral sides of 3 week old wild type or homozygous *ΔHoxd10* host mice, as described previously [57]. Transplanted glands and host controls were harvested on L1 within 12 h of parturition.

All mice used to generate tissue donors had been backcrossed at least eight generations and tested by transplantation to ensure histocompatibility.

### Pituitary Isografts

Pregnant *ΔHoxd10* host mice were transplanted with a single pituitary isograft from a female wild type donor. The pituitary was placed into the kidney capsule of the host mouse or given a control sham operation at P12. The procedure was performed as described previously [58]. At L2, mammary glands were harvested and processed for whole gland and histological analysis.

### Hormone Analysis

Blood was collected and serum was prepared from wild type and *ΔHoxd10* and *ΔHoxd9/d10* homozygous female mice at lactation day two. Samples were run on a mouse prolactin ELISA kit according to protocol (Cat# RAB0408, Sigma-Aldrich). Reading and analysis of the ELISA plate was performed using an iMark microplate reader and Microplate Manager 6 (Bio-Rad). Analysis of progesterone and estradiol serum concentrations was performed by Dr. A. F. Parlow at the National Hormone and Peptide Program, Harbor-UCLA Medical Center, Torrance, CA.

## Electronic supplementary material

Online Resource 1 (**a**) Penetrance of lactation impairment phenotype. a: versus WT: *p* ≤ 0.05; b: versus *ΔHoxd10*: *p* ≤ 0.05; Error bars represent mean ± S.E.M. (**b**) Expressivity of lactation impairment phenotype. Genotype for which the data applies appears to the left of the graph. (**c**) Position-specific frequency of lactation impairment phenotype along the anterior-posterior axis. Genotype for which the data applies appears to the left of the graph (PDF 306 kb)

Online Resource 2 (**a**) QPCR analysis of *Hoxd9* and *Hoxd10* genes in the mouse mammary gland at lactation day 2. a: versus WT: *p* ≤ 0.05; b: versus *ΔHoxd10*: *p* ≤ 0.05; whiskers represent range. (**b**) QPCR analysis of paralogous *Hox9* and *Hox10* genes in the mouse mammary gland at lactation day 2. a: versus WT: *p* ≤ 0.05; b: versus *ΔHoxd10*: *p* ≤ 0.05; whiskers represent range (PDF 120 kb)

Online Resource 3 Serum analysis of estradiol, progesterone, and prolactin and immunohistochemical analysis of phosphorylated STAT5 for pituitary isografts. (**a**) Analysis of serum estradiol at L2. (**b**) Analysis of serum progesterone at L2. (**c**) Analysis of serum prolactin at L2. Error bars represent mean ± S.E.M. (**d**) *ΔHoxd10* homozygote with pituitary isograft sham control. (**d**) *ΔHoxd10* homozygote with sham control procedure. L2. Failed lactation. Reduced phosphorylation of STAT5. Scale bar = 20 μm. (**e**) *ΔHoxd10* homozygote with pituitary isograft. L2. Failed lactation. Phosphorylation of STAT5 is slightly elevated. Inset shows high levels of pSTAT5 in functioning regions (PDF 139 kb)
