# Supplementary figures and images for: Hoxd10 Is Required Systemically for Secretory Activation in Lactation and Interacts Genetically with Hoxd9
Source: J Mammary Gland Biol Neoplasia. 2020 Jul 23;25(2):145–62. doi: 10.1007/s10911-020-09454-3 (PMC7392944; doi:10.1007/s10911-020-09454-3)

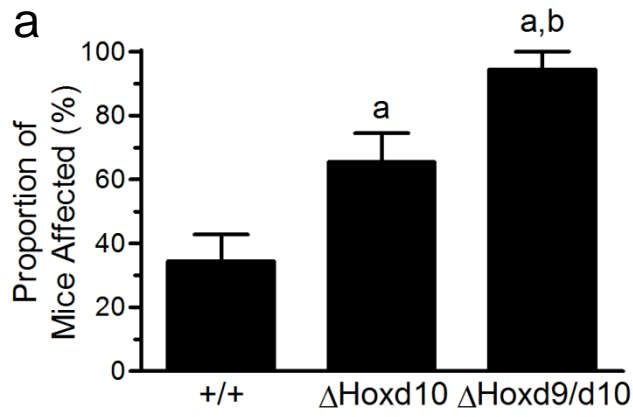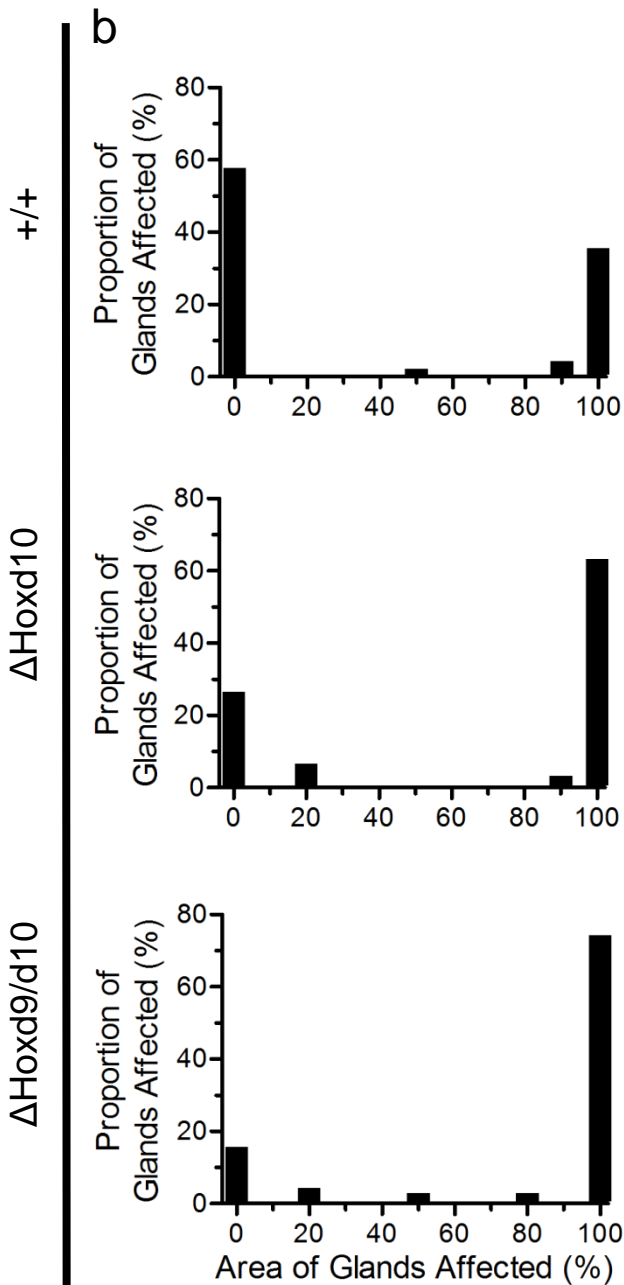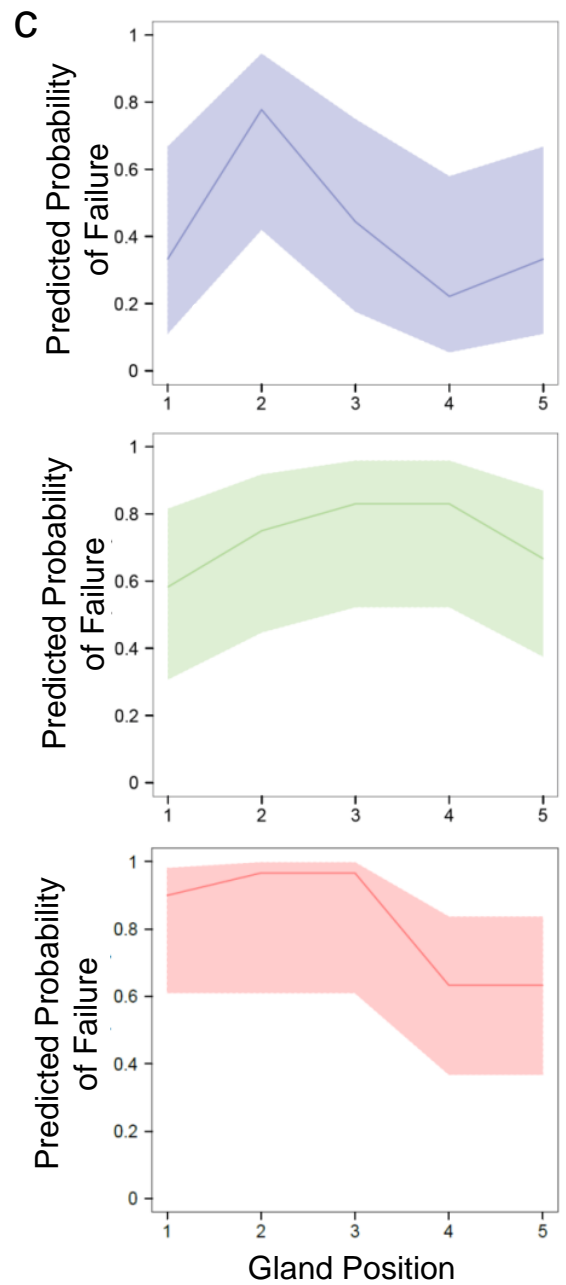

Supplement: Supplementary file 1 — (a) Penetrance of lactation impairment phenotype. a: versus WT: p ≤ 0.05; b: versus ΔHoxd10: p ≤ 0.05; Error bars represent mean ± S.E.M. (b) Expressivity of lactation impairment phenotype. Genotype for which the data applies appears to the left of the graph. (c) Position-specific frequency of lactation impairment phenotype along the anterior-posterior axis. Genotype for which the data applies appears to the left of the graph (PDF 306 kb) [file 10911_2020_9454_MOESM1_ESM.pdf]

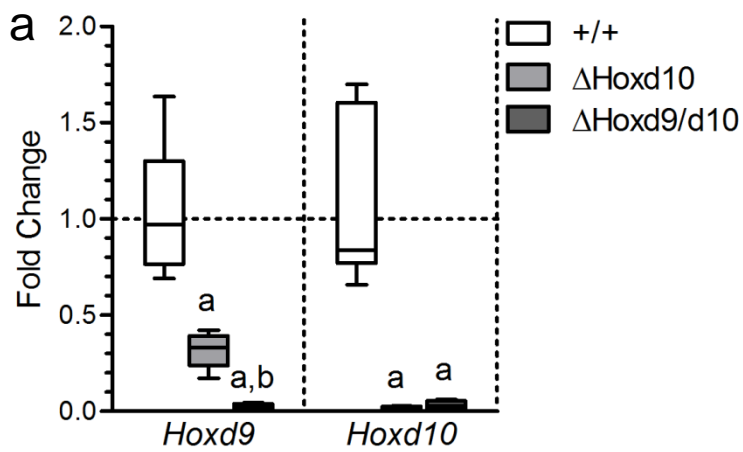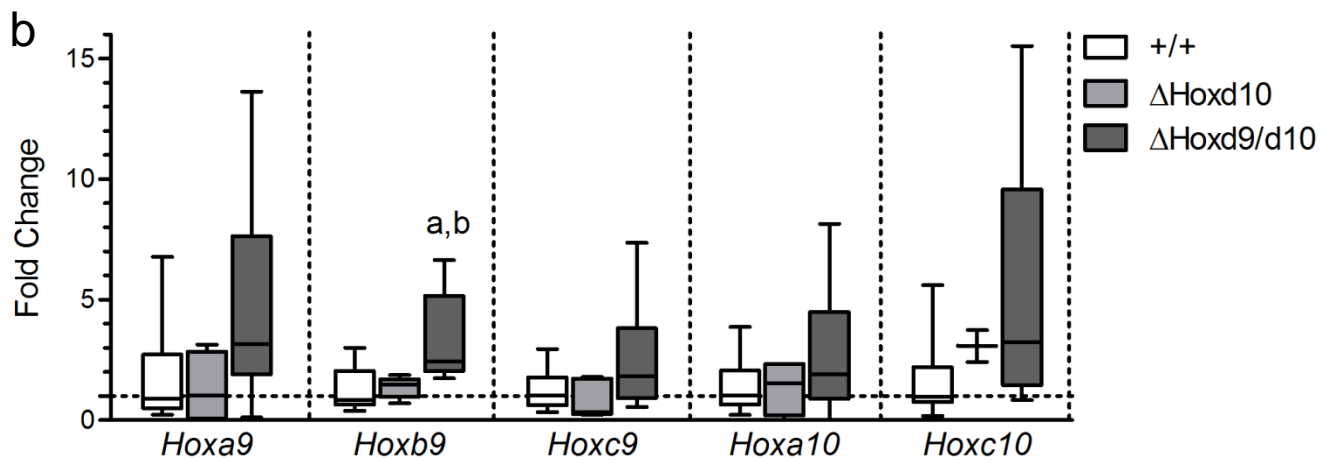

Supplement: Supplementary file 2 — (a) QPCR analysis of Hoxd9 and Hoxd10 genes in the mouse mammary gland at lactation day 2. a: versus WT: p ≤ 0.05; b: versus ΔHoxd10: p ≤ 0.05; whiskers represent range. (b) QPCR analysis of paralogous Hox9 and Hox10 genes in the mouse mammary gland at lactation day 2. a: versus WT: p ≤ 0.05; b: versus ΔHoxd10: p ≤ 0.05; whiskers represent range (PDF 120 kb) [file 10911_2020_9454_MOESM2_ESM.pdf]

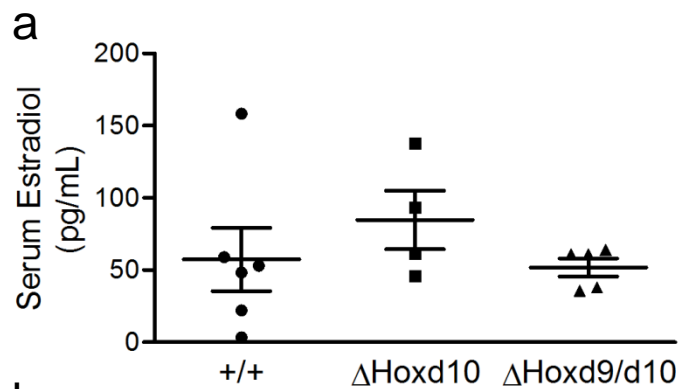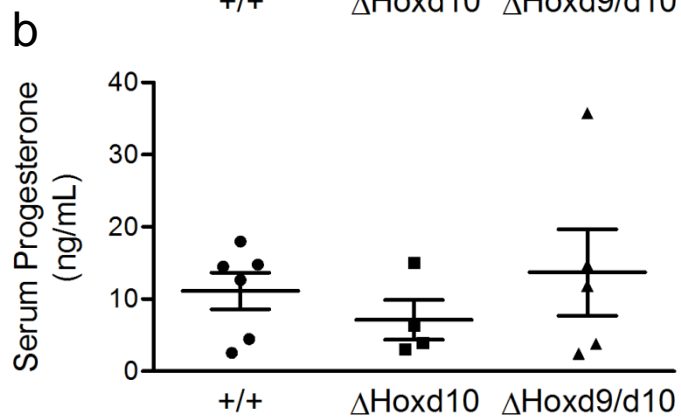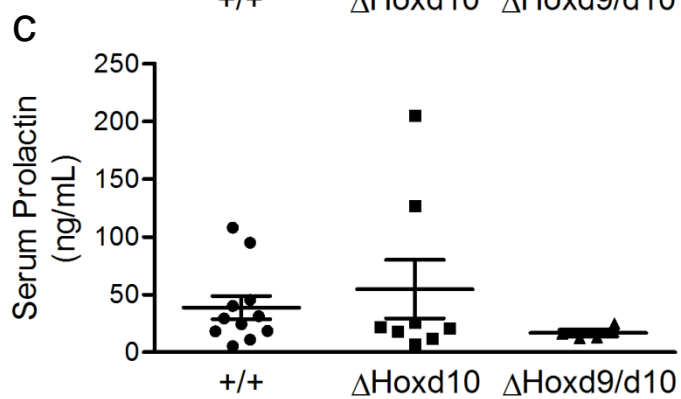

Sham Control

Pituitary Isograft

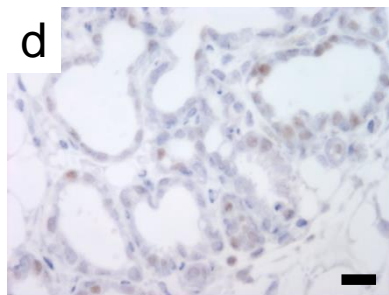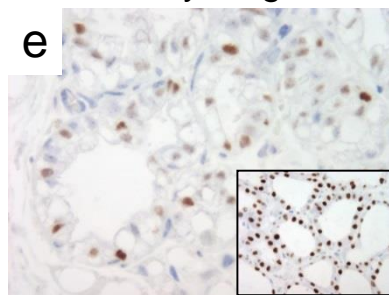

Supplement: Supplementary file 3 — Serum analysis of estradiol, progesterone, and prolactin and immunohistochemical analysis of phosphorylated STAT5 for pituitary isografts. (a) Analysis of serum estradiol at L2. (b) Analysis of serum progesterone at L2. (c) Analysis of serum prolactin at L2. Error bars represent mean ± S.E.M. (d) ΔHoxd10 homozygote with pituitary isograft sham control. (d) ΔHoxd10 homozygote with sham control procedure. L2. Failed lactation. Reduced phosphorylation of STAT5. Scale bar = 20 μm. (e) ΔHoxd10 homozygote with pituitary isograft. L2. Failed lactation. Phosphorylation of STAT5 is slightly elevated. Inset shows high levels of pSTAT5 in functioning regions (PDF 139 kb) [file 10911_2020_9454_MOESM3_ESM.pdf]
